# Natural Orifice Transurethral Endoscopic Vesicovaginal Fistula (NOTE-VVF) Treatment for Early and Small Fistulas: Case Report and the Point of Technique

**DOI:** 10.7759/cureus.23786

**Published:** 2022-04-03

**Authors:** Abdullah Demirtaş, Gökhan Sönmez, Şevket T Tombul, Abdullah Golbasi, Türev Demirtaş

**Affiliations:** 1 Urology, Erciyes University, Kayseri, TUR; 2 Urology, Nevsehir State Hospital, Nevşehir, TUR; 3 History of Medicine and Ethics, Erciyes University, Kayseri, TUR

**Keywords:** natural orifice, transurethral, endoscopic, laparoscopic, vesicovaginal fistula

## Abstract

Minimally invasive surgical approaches have become highly popular in line with technological advancements. In vesicovaginal fistula (VVF) repair, numerous minimally invasive surgical techniques have been described, namely, laparoscopic, robotic, and transvaginal techniques, and used. However, these techniques still require invasiveness. In this report, we present a patient with iatrogenic VVF on whom we applied a novel “zero-incision” technique, Natural Orifice Transurethral Endoscopic Vesicovaginal Fistula (NOTE-VVF) treatment, to repair the fistula tract by advancing the laparoscopic trocar through a natural orifice, i.e., urethra.

## Introduction

Vesicovaginal fistula (VVF) is an abnormal epithelialized or fibrous connection between the bladder and the vagina, resulting in continuous and unremitting urinary incontinence. VVF is rare in developed countries and usually develops secondary to malignancies, radiotherapy, or surgical trauma [1].

The diagnosis of VVF is usually made with a detailed history, outpatient diagnostic tests, and appropriate imaging techniques. The mainstay surgical treatment of VVF is fistula tract repair. However, the surgical technique to be performed is determined based on the location of VVF and the experience of the surgeon [2]. Transvaginal repair, transabdominal repair, and transabdominal transvesical repair are the most commonly preferred techniques in the surgical treatment of VVF [3-5].

Minimally invasive surgical approaches have become highly popular in line with technological advancements. These "zero-incision" approaches provide substantial comfort to both the patient and the surgeons in the postoperative period [6-8]. In 2001, Mack was the first to predict that natural orifices will be frequently used by surgeons in the future and introduced the concept of “Natural Orifice” [6]. In later years, this concept was renamed as Natural Orifice Transluminal Endoscopic Surgery (NOTES) and became applicable in numerous surgical procedures [7,8].

In this report, we present a VVF patient in whom, for the first time in literature, a novel “zero-incision” technique, Natural Orifice Transurethral Endoscopic Vesicovaginal Fistula (NOTE-VVF) treatment, was applied to repair the fistula tract by advancing the trocar through the urethra.

## Case presentation

A 45-year-old female patient was admitted to our clinic with the complaint of continuous urinary incontinence that started immediately after total abdominal hysterectomy that was performed due to uterine myoma in another center about three weeks earlier. Patient history indicated that she had no complaint of urinary incontinence prior to hysterectomy.

**Figure 1:**
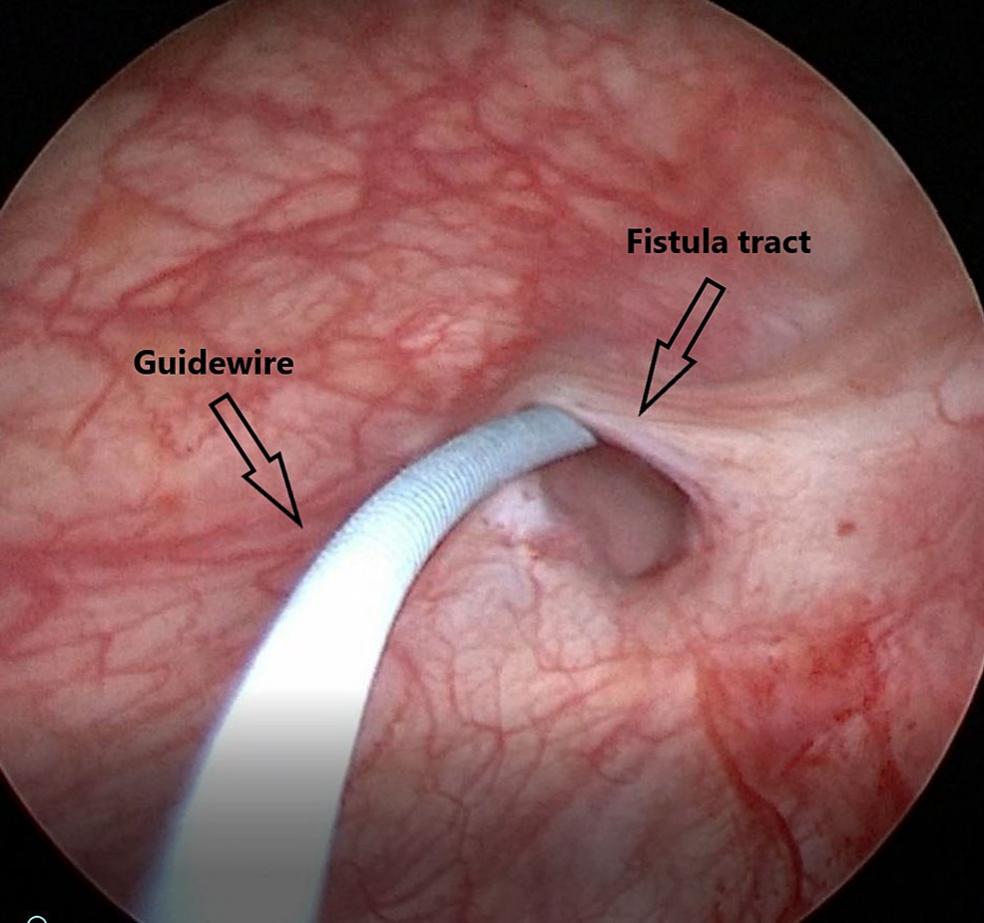
At cystoscopy, the fistula tract and the guide wire inserted through it.

Urogynecological examination indicated urine leakage from the vagina, not from the urethra. Serum creatinine level, complete urinalysis, urinary culture, and in urinary ultrasonography, kidneys, ureters, and bladder were normal. Due to a suspicion of VVF, a 14 Fr urethral catheter (Rusch, Kemen, Germany) was inserted in the bladder and the methylene blue test revealed urinary leakage from the vagina. The diagnosis of VVF was confirmed by the visualization of a fistula tract approximately 5 mm in diameter in the bladder trigone region on cystoscopy which was performed on an outpatient basis (Figure 1). Elective surgery was planned for the fistula repair.

**Figure 2:**
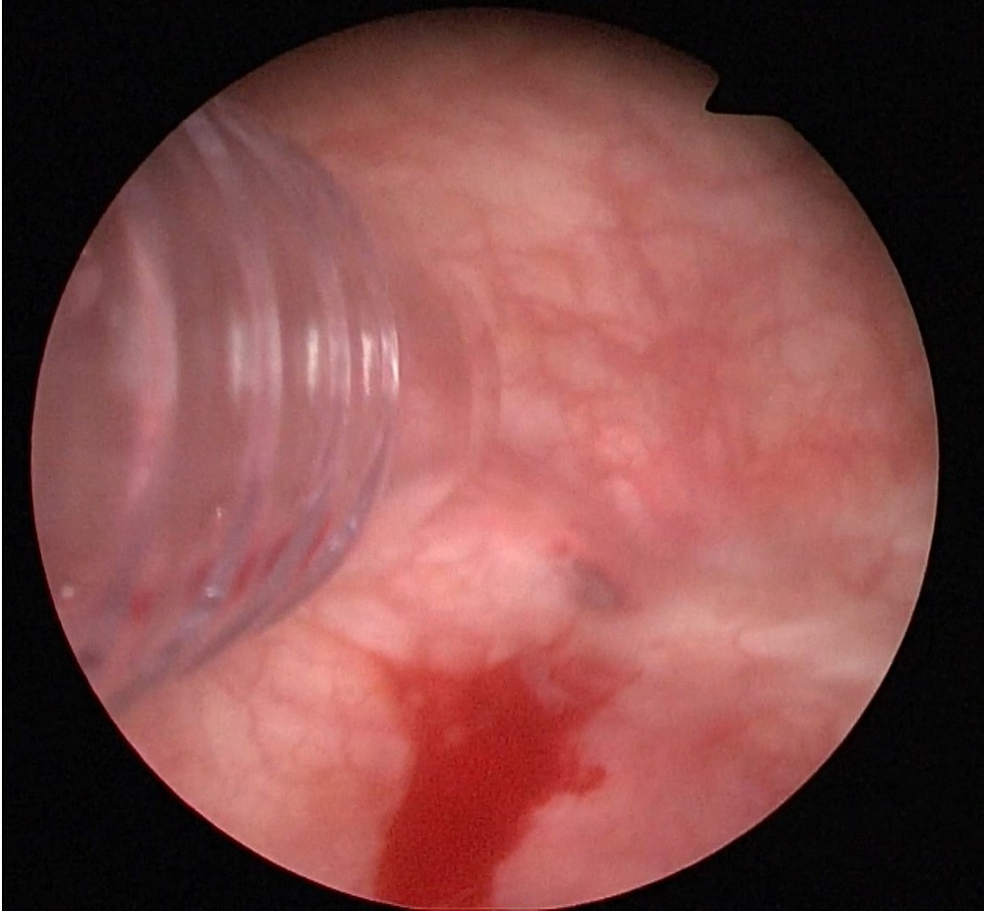
Trocar inserted through the urethra into the bladder from the side of the 12 Fr cystoscope.

**Figure 3:**
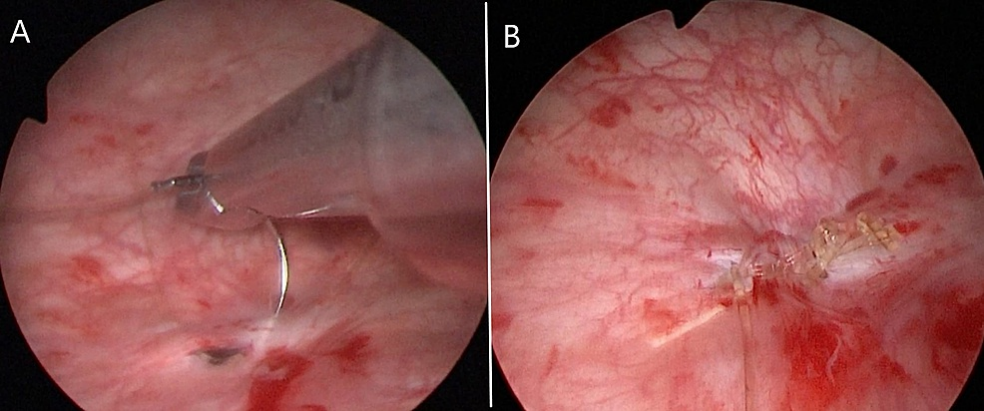
The fistula line was sutured at 1-2 mm intervals and the bladder side was closed using 4/0 absorbable looped v-loc sutures that were advanced into the bladder through the working trocar (A). Appearance of the fistula line after the suturing process is completed (B).

Following preoperative preparation, the patient was transferred to the operating theater and was placed in the lithotomy position at operation room under spinal anesthesia. After cleaning and draping, the location and width of the fistula tract was determined by entering the bladder with the cystoscopy. An optical lens (for imaging) and a 5 mm laparoscopic trocar (working trocar) were carefully inserted into the bladder through the female urethra (Figure 2). Subsequently, the VVF line was sutured at 1-2 mm intervals and the bladder side was closed using 4/0 absorbable loop back v-loc sutures that were advanced into the bladder through the working trocar (Figure 3A and 3B). The procedure was completed after inserting a 14 Fr urethral catheter in the bladder.

**Figure 4:**
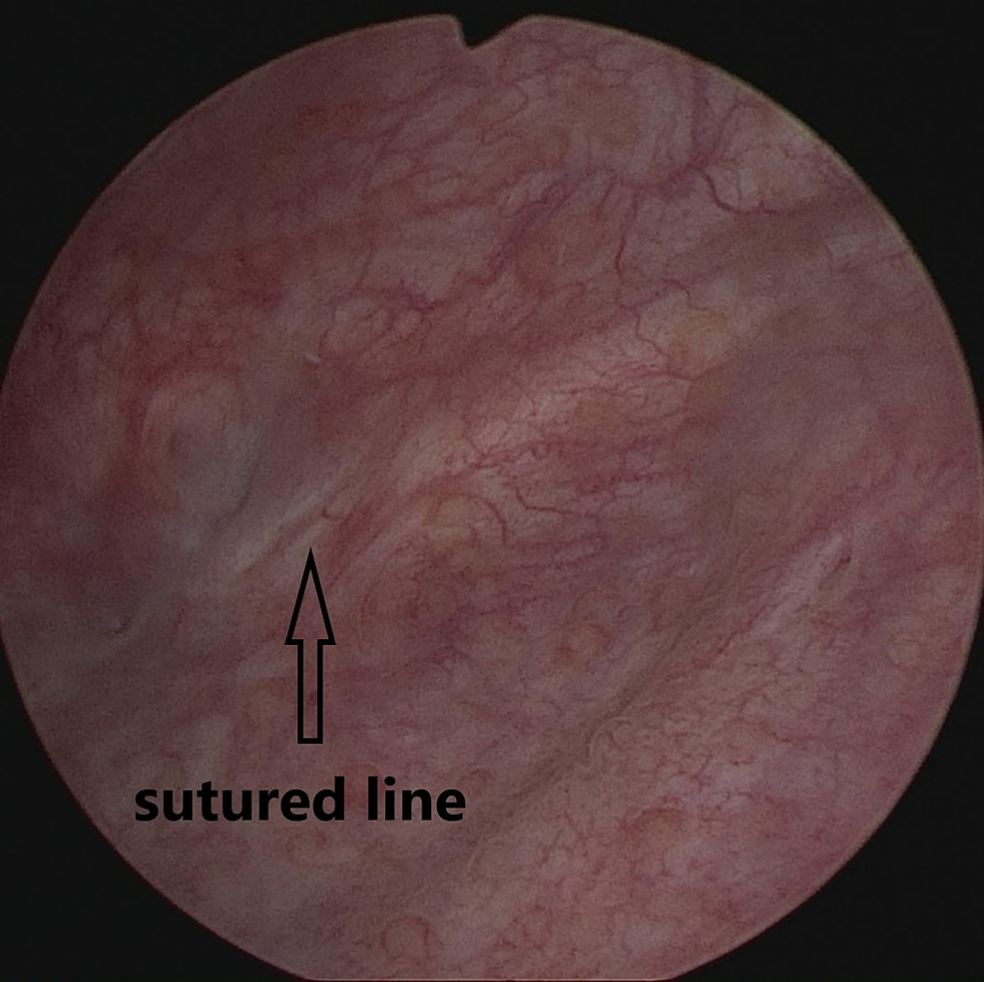
In the postoperative 3rd month control cystoscopy, it was observed that the fistula line was healed.

In this case, VVF repair was performed with the NOTE-VVF treatment technique and the patient was discharged 24 hours after surgery (total operative time: 23 mins). The patient was not given any anticholinergic treatment. On Day 10 after surgery, the patient had no urinary incontinence and no methylene blue leakage from the vagina following the Valsalva maneuver and, thus, the urethral catheter was removed. No urinary incontinence was present at later follow-up visits, and the cystoscopy performed at Month 3 showed complete closure of the fistula tract (Figure 4). No peri- or post-operative complication occurred in the patient.

## Discussion

Hysterectomy is the most common iatrogenic cause of VVF in developed countries. In the literature, VVF treatment is primarily based on case reports or series documenting personal experiences of urologists and gynecologists [2-5]. Transvaginal repair, transabdominal repair, and transabdominal transvesical repair are the most commonly preferred techniques in surgical treatment of VVF, among which transvaginal repair is the most common. Transabdominal repair, on the other hand, is often preferred in patients who cannot lie in the lithotomy position or in patients with more complicated conditions such as a history of radiotherapy [2].

In the case presented, we applied the NOTE-VVF technique for the first time in the literature and a small acute-stage VVF was successfully repaired using a minimally invasive surgical technique within a short period of time. The common view on the timing of VVF repair recommends performing early repair (within the first three weeks) or postponing the repair to three months later [9]. In our patient, we preferred early repair and performed the VVF repair within the first three weeks, mainly due to the need to repair the fistula tract before epithelialization. As is commonly known, an epithelialized fistula tract is unlikely to be closed by primary repair and, thus, more invasive interventions requiring removal of the fistula tract may be needed in the future.

Literature indicates that small fistulas, though rarely, can close spontaneously with good bladder drainage [10,11]. Thompson et al. advocated that 15-20% of simple and small fistulas may heal spontaneously after bladder drainage [10]. Due to this low probability, preoperative conservative treatment was not performed in our patient to reduce the possibility of epithelialization of the fistula tract.

Regardless of the technique applied, the most important issue in VVF repair, as suggested in the literature, is achieving adequate bladder drainage after surgery to allow tissue healing [11]. Accordingly, in our patient, the urethral catheter was maintained in the bladder for 10 days postoperatively and was removed after the methylene test to ensure complete closure of the fistula tract.

The advantages of the NOTE-VVF technique include no incision, application with local anesthesia, absence of blood loss, and shorter operative time and postoperative hospitalization period. One of the most important advantages of the technique is that even if the treatment is unsuccessful, it does not pose an obstacle and difficulty for other invasive surgical procedures. However, the technique may be disadvantageous as well since it can only be applied in small and simple fistulas and requires early fistula repair (within the first three weeks if possible) due to the risk of epithelialization in the fistula tract. Additionally, despite the lack of substantial evidence, it is tempting to consider that the success rate of this technique is likely to be lower in fistulas associated with radiotherapy or malignancy.

## Conclusions

We consider that the NOTE-VVF technique introduced in the present study, which can be applied by entering the bladder through the urethra only by using 5 mm laparoscopic trocar and a thin optical lens, can be successfully used in the treatment of small, non-complicated, acute-stage, iatrogenic VVFs. The fact that this method can be performed with minimally invasive surgical intervention can be a testable treatment step before moving on to more complex surgical treatments. Further longitudinal studies with larger patient series are needed to verify the utility of this technique.
